# Useless Disinfection

**Published:** 1924-02

**Authors:** 


### Useless Disinfection.

SIR,—I had occasion recently to refer to the last Annual Report of the Chief Medical Officer of the Ministry of Health, and I came across the following interesting passage under the heading of "Scarlet Fever":—

> Increasing knowledge in regard to the transmission of infection from scarlet fever cases such, for example, as has been obtained by studies of cases treated in hospital on the "barrier" system, leads to doubt whether articles of furniture, bedding and clothing, or the walls and floors of rooms in houses where scarlet fever has occurred, are likely to play more than a practically negligible part in the transmission of this disease.

Some of us have felt an intense horror at being brought within a hundred yards of clothing or furniture which have been in the same room as a patient suffering from infectious disease. We have been profoundly anxious until it has been disinfected or, better still, destroyed. Is it really a fact that we have been worrying ourselves unnecessarily all the time? Sir George Newman promises to return to the subject in his next report. In the meantime it would be interesting to have the views of some Medical Officers of Health on the question.

Coming so soon after the recent statement attributed in your December issue to Dr. Wynne, M.O.H., for Sheffield, that there is no discoverable danger to health in an escape of gas from a drain or sewer, it would seem that a new world is opening up in which we shall be able to walk free from the attentions of microbes and inspectors.

ENQUIRER.

[There is a reference to the matter in the Report in this issue of our Special Commissioner's interview with the Brighton Health Authority.—*Ed.* HOSPITAL AND HEALTH REVIEW.]

Every kind of surgical appliance is included in the new catalogue of the Surgical Manufacturing Co., Ltd., and, with few exceptions, all are made at the firm's London factories under expert supervision. The company's address is 83-85, Mortimer Street, W. 1, and it supplies surgical instruments to the Admiralty, War Office, India Office, the French Government, the American Government, etc. Members of the medical and nursing profession and hospital secretaries are invited to inspect the various articles in course of construction, and, being actual manufacturers, the company is able to offer goods of excellent quality at competitive prices.

